# The R72P P53 mutation is associated with familial breast cancer in Jewish women

**DOI:** 10.1038/sj.bjc.6602451

**Published:** 2005-03-01

**Authors:** T Ohayon, R Gershoni-Baruch, M Z Papa, T Distelman Menachem, S Eisenberg Barzilai, E Friedman

**Affiliations:** 1 The Susanne Levy Gertner Oncogenetics Unit, The Danek Gertner Institute of Genetics, Chaim Sheba Medical Center, Tel-Hashomer 52621, Israel; 2 The Genetics Institute, Rambam Medical Center, Haifa, Israel; 3 The Bruce Rappaport School of Medicine, Technion, Haifa, Israel; 4 Department of Oncological Surgery, Chaim Sheba Medical Center, Tel-Hashomer, Israel; 5 The Sackler School of Medicine, Tel-Aviv University, Ramat Aviv Tel Aviv, Israel

**Keywords:** *P53* gene, inherited predisposition to breast cancer, DGGE, Arg72Pro mutation, germline mutations

## Abstract

*BRCA1/BRCA2* mutations account for a substantial proportion of familial breast cancer, but clearly mutations in additional genes exist, one candidate being the *p53* gene. To evaluate its putative involvement in inherited predisposition to breast/ovarian cancer in Jewish high-risk women, mutational analysis of the *p53* gene (exons 4–9) was carried out using exon-specific polymerase chain reaction followed by denaturing gradient gel electrophoresis (DGGE) analysis, complemented by DNA sequencing of abnormally migrating fragments. Overall, 132 Jewish breast cancer patient non-*BRCA1/2* mutation carriers and 167 average risk controls (Ashkenazi (*n*=60), non-Ashkenazi (*n*=107)) were genotyped, and no inactivating p53 germline mutations were detected. Consistent migration abnormalities were noted in 167 fragments, 134 of which were shown to be the Arg72Pro polymorphism, whereas migration abnormalities in fragments containing exons 4 (*n*=2) and 6 (*n*=23) and introns 3 (*n*=4) and 9 (*n*=4) corresponded to five previously described polymorphisms. Allele distribution of the R72P missense mutation between ethnically diverse Jewish breast cancer cases and average risk controls showed significant differences: among non-Ashkenazi breast cancer cases, 62.5%, 33.3% and 4.2% were homozygous, heterozygous and homozygous for the Arg72, Arg72Pro and the Pro72 polymorphism, respectively, whereas for controls, the distribution was 22.4%, 65.4% and 12.2%, respectively (*P*=0.00052), and among Ashkenazi breast cancer cases, allele distribution was 68.5%, 29.6% and 1.9%, whereas for controls, the distribution was 50%, 40% and 10%, respectively (*P*=0.0125). We conclude that arginine homozygosity at codon 72 of the *p53* gene is associated with a significant increased breast cancer risk in Jewish high-risk population.

Germline mutations in the *BRCA1* (MIM#113705) and *BRCA2* (MIM#600185) genes account for genetic predisposition and increased risk for breast and ovarian cancer in the majority of families with inherited predisposition to both these neoplasms and in 20–40% of families with site-specific breast cancer (Hall *et al*, 1990; Easton *et al*, 1993; Frank *et al*, 1998). In the majority of high-risk families of different ethnicities, germline mutations are scattered throughout both genes and are family specific (BIC database). In Jewish individuals, only a handful of recurring mutations have been described in high-risk families (Ganguly *et al*, 1997; Shiri-Sverdlov *et al*, 2000). Notably, among Ashkenazim (East European ancestry), three mutations in *BRCA1* (185delAG, 5382InsC) and *BRCA2* (6174delT) predominate, and can be detected in more than 80% of familial breast and ovarian cancer, in about 40–50% of site-specific familial breast cancer and in 2.5% of the general population of this ethnic group (Abeliovich *et al*, 1997). Among non-Ashkenazi (Sephardic) Jews, the 185delAG and the Tyr978X *BRCA1* and the 8765delAT in *BRCA2* mutations can be detected in high-risk families (Shiri-Sverdlov *et al*, 2001). Yet, a substantial proportion of familial breast cancer cases have no identifiable *BRCA1/BRCA2* germline mutations (Tereschenko *et al*, 2002; Perkowska *et al*, 2003), and other, yet unidentified genetic factors underlie these cases (Slattery and Kerber, 1993). One of the most promising candidates as target for molecular analysis as an inherited breast cancer predisposition gene is the *p53* tumour suppressor gene: it is frequently somatically mutated in a wide range of human cancers including breast cancer (Harris and Hollstein, 1993); germline mutations lead to an increased risk for developing diverse malignancies, including 25–30% of breast cancer cases (Bukholm *et al*, 1997), in the context of Li–Fraumeni syndrome (LFS) or LFL – Li–Fraumeni-like syndrome (Varley, 2003). In addition, based on its pivotal role in DNA damage repair and its physical and functional interactions with BRCA1 and BRCA2 proteins (Storey *et al*, 1998), p53 seems to be a strong candidate breast cancer predisposition gene. However, previous analyses of high-risk families of different ethnic background yielded a paucity of germline mutations in *p53* gene in familial breast cancer (Zelada-Hedman *et al*, 1997; Balz *et al*, 2002).

In addition to *bona fide* inactivating mutations, which seem to cluster to the central region of the gene (exons 5–8) (Hartmann *et al*, 1995), there are several missense mutations within this gene that seem to have a deleterious effect on p53 function (p53 database). Notably, the Pro72 and Arg72 variants have been reported to differ in functional activity: the Arg72 variant was found to be more susceptible to degradation by the human papillomavirus (HPV) E6 type 18 protein (Storey *et al*, 1998), while Pro72 is a stronger inducer of transcription than Arg72. Additionally, the Arg72 variant suppressed effectively cellular transformation (Thomas *et al*, 1999), and was more efficient than the Pro72 variant at inducing apoptosis (Dumont *et al*, 2003).

In the present study, the putative contribution of *p53* germline mutations to inherited predisposition to breast/ovarian cancer was assessed, initially by genotyping high-risk Jewish individual noncarriers of the predominant Jewish *BRCA1/2* mutations for *p53* germline mutations, and subsequently by comparing the distribution of the R72P alleles among cases and controls.

## MATERIALS AND METHODS

### DNA isolation

Genomic DNA was prepared from anticoagulated venous blood samples, using standard techniques, and using the PUREGene DNA extraction kit (Gentra Systems Inc., Minneapolis, MN, USA) and following the manufacturer's recommended protocol.

### Breast cancer cases

Ashkenazi and non-Ashkenazi individuals who were counselled at the Oncogenetic services of the Sheba and Rambam Medical Centers were eligible for participation. Their ethnic origin was determined by an interview, and dating parental origin as far back as possible by at least three generations. All individuals had breast cancer and one of the following additional criteria: ovarian cancer; two first or second degree relatives with one of these neoplasms at any age; age at onset under 40 for breast cancer, bilateral breast cancer; cooccurrence of breast and ovarian cancer in one first-degree relative. The study was approved by the Institutional review board of both participating medical centers (Rambam and Sheba) and each participant signed a written, informed consent. All participants were tested, and found not to harbour any of the predominant Jewish mutations in BRCA1/BRCA2 (see below).

### Controls

Jewish individuals of diverse ethnic origin who came for genetic counselling at the Genetics institutes in Sheba and Rambam Medical Centers served as ‘ethnic controls’. These individuals were either counselled for prenatal disorders or were counselled as to their risk for developing cancer, and none was deemed ‘high cancer risk’ by standard criteria. All tested individuals were unrelated to each other, had no personal or relevant family history of breast or ovarian cancer, and their precise ancestry was confirmed at least three generations back.

### Mutation analyses of the predominant Jewish mutations in BRCA1 and BRCA2

Mutational analyses for the three predominant mutations (185delAG, 5382InsC in *BRCA1* and 6174delT in *BRCA2*) were carried out by restriction enzyme digest of amplified polymerase chain reaction (PCR) product. The primers used generated novel restriction sites that distinguish the mutant from the wild-type allele. Thus, restriction enzyme digest followed PCR, and analysis of the digested PCR products on agarose gels was carried out as described previously (Abeliovich *et al*, 1997; Rohlfs *et al*, 1997). The Tyr978X mutation was detected by a modified PCR-restriction enzyme digest with *Eco*RV, as described previously (Theodor *et al*, 1998). With each gel, a positive control (i.e. a known mutation proven by DNA sequencing) was run in an adjacent lane.

## MUTATIONAL ANALYSES OF THE *P53* GENE

### Polymerase chain reaction

Polymerase chain reactions were performed in a final volume of 50 *μ*l containing 3 *μ*l template DNA (about 50–100 ng), 10 pM of each primer, 200 mM of each dNTP, standard PCR buffer (1.5 mM MgCl_2_), 1 U of Taq DNA polymerase. Thermal cycling was accomplished by PTC-100-60 thermocycler (MJ Research Inc., Watertown, MA, USA). The cycling profile included an initial denaturation at 94°C for 5 min, followed by 35 cycles of thermal cycling including 94°C for 20 s, the designated annealing temperature (range 52–68°C) for 1 min, extension at 72°C for 20 s and a final extension cycle at 72°C for 5 min.

### Denaturing gradient gel electrophoresis (DGGE) and sequence analysis of the *p53* gene

Primer sequences, PCR conditions and DGGE analyses parameters were carried out under the conditions described previously (Guldberg *et al*, 1997). All consistently abnormally migrating fragments (i.e. repeated abnormalities on three independent PCRs) were subject to sequence analysis using the big Dye terminator chemistry and kit (PE Biosystems, Foster City, CA, USA), and using the ABI Prism 310 semiautomatic DNA sequencer (PE Biosystems).

### Statistical analyses

*χ*^2^ was performed in order to evaluate Hardy–Weinberg equilibrium (HWE) in the control group, alleles's prevalence in ethnicities, association genotype/allele–phenotype, Hardy–Weinberg deviation (in the patient group), Fisher's exact test and Armitage's trend test (Crow, 2001). Type I error was set at 5%, as is the accepted practised level.

The websites for checking HWE in the control group, *χ*^2^ for genes and alleles association, HWE by the phenotypic categories in the patient group and Armitage's trend test: http://ihg.gsf.de/cgi-bin/hw/h wa1.pl, http://www.unc.edu/~preacher/c hisq/chisq.htm.

## RESULTS

### Characteristics of breast cancer cases

Overall, 132 Jewish breast cancer patients were analysed: 108 (82%) patients were of Ashkenazi origin and 24 (18%) were of non-Ashkenazi origin. Among individuals of Ashkenazi origin, 10 patients presented with bilateral breast cancer (9.25%); age range at diagnosis of first breast cancer was 23–70 years (46±10.1 years – mean±s.d.); mean age at counselling was 50.2±10.8 years; the interval between date of diagnosis and date of testing was 0.5–27 years (5.35±5.8 years).

Among non-Ashkenazi cases (*n*=24), three patients presented with bilateral breast cancer (12.5%), and the age range at diagnosis of first breast cancer was 26–61 years (42.6±9.8 years); mean age range at counselling was 49±10.6 years. The interval between date of diagnosis and date of testing ranged from 0.5 to 33 years (6.6±7.6 years).

### Mutational analyses of the predominant *BRCA1*/*BRCA2* Jewish mutations among cases

All cases were tested and found not to be carriers of one of the predominant mutations in *BRCA1* (185delAG, 5382InsC) and *BRCA2* (6174delT). The non-Ashkenazis were also analysed for the Tyr978X *BRCA1* mutation, and none was a carrier.

### Controls

The age range among Ashkenazi controls (*n*=60) was 27–65 years (39.3±8.8 years).

The age range among non-Ashkenazi controls (*n*=107) was 21–70 years (34.1±9.6 years).

### Mutational analysis

DGGE analysis of exons 4–9 of the *p53* gene revealed consistent migration abnormalities in 167 fragments; 134 of these fragments were shown to be the Arg72Pro polymorphism (see below). Migration abnormalities in other fragments containing exons 4 and 6 and introns 3 and 9 were detected in 2, 23, 4 and 4 patients, respectively. All abnormally migrating fragments were sequenced and results showed that all abnormal migration patterns could be attributed to previously described polymorphisms (Table 1). No inactivating mutations could be shown in any of the analysed individuals.

#### R72P analysis

The distribution of the Arg72Pro polymorphism in cases and controls is presented in Tables 2 and 3. Among cases, no deviation from the HWE was shown. Allele distribution of the R72P missense mutation among breast cancer cases (Ashkenazi and non-Ashkenazi) and average risk controls (Ashkenazi (*n*=60), non-Ashkenazi (*n*=107)) showed significant differences: among non-Ashkenazi cases, 62.5%, 33.3% and 4.2% were homozygous, heterozygous and homozygous for the Arg72, Arg72Pro and the Pro72 polymorphisms, respectively, whereas for controls the distribution was 22.4%, 65.4% and 12.2%, respectively (*P*=0.00052), and among Ashkenazi cases, allele distribution was 68.5%, 29.6% and 1.9%, whereas for controls, the distribution was 50%, 40% and 10%, respectively (*P*=0.0125) (Tables 2 and 3).

In Armitage's trend test, which examines genotype–phenotype correlation, Arg72 homozygosity was significantly associated with breast cancer cases for the Ashkenazi subgroup (*P*=0.00452) as well as in the non-Ashkenazi subgroup (*P*=0.0075).

Analysis by allele prevalence (A/P) also shows a significant association between the arginine-bearing allele and high-risk cases (*P*=0.00432 for Ashkenazi origin; *P*=0.02412 for non-Ashkenazi origin).

## DISCUSSION

In the present study, no inactivating germline mutations in exons 4–9 of the *p53* gene were detected in high-risk Jewish individuals who are not carriers of any of the predominant Jewish mutations in BRCA1/BRCA2. Previous analyses of high-risk families of different ethnic background yielded a paucity of germline mutations in *p53* gene in familial breast cancer: only two mutations were identified among 237 high-risk Norwegian individuals (Borresen *et al*, 1992), one mutation among 126 high-risk American individuals (Sidransky *et al*, 1992) and one mutation among 21 families from the UK (Evans *et al*, 2002). Similar to the results of the present study, no inactivating p53 germline mutations were found in any of Swedish familial breast cancer patients (Zelada-Hedman *et al*, 1997) or in German breast and/or ovarian cancer families (Balz *et al*, 2002). Thus, despite features suggesting that this might be a strong candidate underlying inherited breast cancer predisposition, p53 germline mutations probably contribute little to these families, unless it is in the context of LFS or LFS-like phenotype. It is still possible that germline sequence alterations in the *p53* gene affect breast cancer risk. Intronic variants within the *p53* gene have a functional effect on p53 protein activity and occur at high frequency in familial breast cancer cases. Lehman *et al* (2000) detected an intronic variant (13964G/C) at a significantly high frequency among familial breast cancer cases (*n*=42), with evidence that this sequence variant is associated with prolonged *in vitro* survival in response to cisplatinum treatment and showed decreased chemotherapy-induced apoptosis (Lehman *et al*, 2000). The data regarding the high rate of this specific polymorphism in high-risk women could not be reproduced in Australian women (Marsh *et al*, 2001). In addition, intronic polymorphisms with no known functional consequences (e.g. intron 3–16 bp insertion) were reported to be over-represented in Swedish (Sjalander *et al*, 1996) and German breast cancer-prone families (Wang-Gohrke *et al*, 1998).

An intriguing finding in the present study is that the distribution of the R72P missense mutation significantly differs in affected individuals compared with ethnically matched controls. Specifically, ethnically diverse Jewish breast cancer individuals are significantly more likely to be R72 homozygotes, compared with average risk, unaffected individuals.

The Pro72 and Arg72 *p53* gene variants have been reported to display different functional activities: the Arg72 variant is more susceptible to degradation by the HPV E6 type 18 protein (Storey *et al*, 1998), while Pro72 is a stronger inducer of transcription than the Arg72 variant. Additionally, the Arg72 variant suppressed effectively cellular transformation (Thomas *et al*, 1999), and was more efficient than the Pro72 variant in inducing apoptosis (Dumont *et al*, 2003).

The rate of this sequence variant has also been evaluated in breast cancer cases in ethnically diverse populations, with inconsistent results. In line with the findings in the present study, a significantly higher prevalence of homozygosity for the p53 arginine-bearing allele was observed in Turkish breast cancer patients compared with controls (Buyru *et al*, 2003), whereas among Japanese breast cancer cases, Pro72 homozygosity was significantly more prevalent than in controls (32% Pro72 in cases and 40.4% in controls) (Huang *et al*, 2003). Notably, there was no evidence of association between p53 codon 72 polymorphism and breast cancer risk Tunisian (Mabrouk *et al*, 2003), and Russian individuals (Suspitsin *et al*, 2003). In addition, this polymorphic variant was reported to be associated with breast cancer survival among English breast cancer patients, but this association lacked statistical significance in multivariate analysis (Goode *et al*, 2002).

The limitations of this study should be pointed out. This is a limited study encompassing only women affected with breast cancer, counselled and tested in two medical centres in Israel. The implications to other breast cancer populations, even among Jewish individuals, are unclear and need to be validated. Furthermore, the applicability of this finding to average risk population is unclear and needs to be established.

In conclusion, the contribution of p53 germline mutations to inherited predisposition to breast cancer by inactivating mutations seems to be limited in Jewish women. It seems that a common missense mutation (R72P) confers an increased risk to breast cancer in familial cases of Jewish origin. The applicability and the generalisability of this preliminary finding need to be confirmed.

## Figures and Tables

**Table 1:** Polymorphisms detected in the *p53* gene

**Ethnic origin**	**Number of cases**	**Exon/intron**	**Sequence alteration**	**Predicted effect on protein**
Ashkenazi	2	Exon 4	c.108G>A	P36P
Ashkenazi	4	Intron 3	Inv3–29	—
Ashkenazi	5	Exon 6	c.606T>C	R202R
Ashkenazi	18	Exon 6	c.639A>G	R213R
Ashkenazi	4	Intron 9	Inv9–13	—

**Table 2:** P53 Arg72Pro polymorphism distribution among non-Ashkenazi Jews, breast cancer cases and controls

	**Cases**		**Controls**				
**P53 alleles**	***n*=24**	**%**	***n*=107**	**%**	**Alleles**	**Genotype**	**Armitage's trend test**
Arg/Arg	15	62.5	24	22.4	*P*=0.02412	*P*=0.00052	*P*=0.00750
Arg/Pro	8	33.3	70	65.4			
Pro/Pro	1	4.2	13	12.2			

**Table 3:** P53 Arg72Pro polymorphism distribution among Ashkenazi Jews, breast cancer cases and controls

	**Cases**		**Controls**				
**P53 alleles**	***n*=108**	**%**	***n*=60**	**%**	**Alleles**	**Genotype**	**Armitage's trend test**
Arg/Arg	74	68.5	30	50	*P*=0.00432	*P*=0.0125	*P*=0.00452
Arg/Pro	32	29.6	24	40			
Pro/Pro	2	1.9	6	10			
